# Development of a Flavor Fingerprint Using HS-GC-IMS for Volatile Compounds from Steamed Potatoes of Different Varieties

**DOI:** 10.3390/foods12112252

**Published:** 2023-06-02

**Authors:** Hong Jiang, Wensheng Duan, Yuci Zhao, Xiaofeng Liu, Guohong Wen, Fankui Zeng, Gang Liu

**Affiliations:** 1 Research & Development Center for Eco-Material and Eco-Chemistry, Lanzhou Institute of Chemical Physics, Chinese Academy of Sciences (CAS), Lanzhou 730000, China; jianghong@licp.cas.cn (H.J.); gangliu@licp.cas.cn (G.L.); 2 College of Life Science and Engineering, Lanzhou University of Technology, Lanzhou 730050, China; 3 Potato Research Institute, Gansu Academy of Agricultural Sciences, Lanzhou 730070, China

**Keywords:** steamed potatoes, varieties, volatile compounds, fingerprint, HS-GC-IMS

## Abstract

The variations in flavor substances across different varieties of steamed potatoes were determined by headspace-gas chromatography ion mobility spectrometry (HS-GC-IMS) combined with sensory evaluation. Results showed that 63 representative compounds, including 27 aldehydes, 14 alcohols, 12 ketones, 4 esters, 2 furans, 1 acid and others, together acted as contributors to the flavors in steamed potatoes. Analysis found that species and concentrations of aldehydes, alcohols and ketones in six varieties were the most abundant. In addition, esters, furans and acid were also responsible for flavor. PCA results showed that volatile compounds in Atlantic, Longshu No. 23, Longshu No. 7 and Longshu No. 14 were similar, while Russet Burbank and Longshu No. 16 had distinct characteristic volatiles, which was consistent with sensory evaluation. The combination of sensory evaluation and HS-GC-IMS provided useful knowledge for charactering volatile compounds of steamed potatoes from different varieties, and also demonstrated the promising application of HS-GC-IMS in the detection of potato flavor with various cooking methods.

## 1. Introduction

With large scale production and consumption, potatoes became the most consumed and economically essential agricultural product prevailing all over the world [1]. In other words, potatoes play a vital role in providing nutrition for human health and ensuring food security. The common approaches for preparing fresh potatoes are by boiling, baking or frying them, according to dining habits under different cultures [2]. In addition, steaming potatoes is another manner of cooking in domestic repast and the catering industry [3]. Among them, the boiled and steamed potatoes are both cooked with the aid of heated water. However, there are subtle differences in the thermal processes of boiling and steaming. Boiled potatoes are processed in the heated water medium, whereas the steamed potatoes are cooked by hot steam. During boiling, a portion of components, including starches, minerals such as P and Fe and soluble vitamins from potatoes, will dissolve in the heated water, which causes a serious nutrition and flavor loss for the potato [4,5]. Instead, the nutrition components and sensory quality of steamed potatoes can be preserved very well. Therefore, steamed potatoes are more preferable in contrast to boiled ones.

To a large extent, the overall sensory characteristics of food are always affected by flavor [6]. Nowadays, with higher rates of self-sufficiency because of economic wealth, flavor preference is a more desirable criterion rather than taste or aroma when people buy food products. The flavor even becomes an essential breeding target in some countries, and this tendency has been conspicuous in multiple crops, especially in potato [3]. Flavor is a perceptual and comprehensive property determined by evaluating the aroma, taste and others sensory attributes [7]. A wide range of volatile compounds, including aldehydes, alcohols, ketones, esters, acids, furans, pyrazines, ethers and so forth, play an important role in potato flavor. As is well-known, a limited amount of volatiles are present in raw tubers, but more volatile compounds will be produced during cooking, with a thermal result for a series of precursors, such as sugars, proteins, lipids and vitamins [8,9]. The chemical reactions that are responsible for flavor compounds production during cooking mainly refer to the Maillard reaction, lipid oxidation and Strecker degradation [10,11,12]. Although several publications have pointed out that some cooking methods, such as boiling, baking or frying, can alter potato flavor due to the changes in flavor compounds [13], there are few studies about potato flavor being affected by steaming. In addition, the genotype is also a direct factor that affects potato flavor. Jitsuyama et al. (2009) demonstrated that the flavors of thirteen potato varieties were significantly different due to the endogenous components and cell morphological traits after steaming [3]. In their research, flavor was only evaluated via tasting sweetness, smoothness and deliciousness, without instrumental analysis. Therefore, it is necessary to analyze the detailed volatile compounds in steamed potatoes among different varieties in combination with sensory evaluation and instrumental analysis.

In the past, the identification and quantitation techniques used for potato flavor have included gas chromatography-mass spectrometry (GC-MS), gas chromatography-offactometry-mass spectrometry (GC-O-MS) and metal-oxide semiconductors such as electronic nose [14]. However, GC-MS needs complicated pre-treatments and is constrained in distinguishing isomeric molecules, GC-O-MS requires significant amounts of repetitive, time-consuming labor, and electronic nose is greatly influenced by environmental factors, leading to the unstable results or poor repeatability. In order to avoid the limitations of complicated pre-treatments, lower detection sensitivity and longer detection time, and to provide an efficient and rapid detection for researchers [13], in recent years, a novel and powerful device, gas chromatography (GC) coupled with ion mobility spectrometry (IMS), has been chosen to accurately test flavor compounds in various foods. It was acknowledged that it has the advantages of ultra-high sensitivity and speed, simplicity, inexpensive cost and easy operation [15]. It should be noted that GC-IMS is operated under ambient atmospheric pressure and temperature and used to analyze trace ionized substances on the basis of their migration in an electric field [16]. HS-GC-IMS have provided convenience to researchers for a vast array of volatile compounds analysis in solid or liquid samples [17]. Therefore, this technique can also be used for volatile flavor analysis in cooked potatoes.

Up to now, most studies have been focused on the flavor of baked, boiled or fried potatoes from different varieties [2,9,18,19,20]. However, to date, few studies have monitored the flavor change in steamed potatoes from diverse varieties. In addition, the published information has revealed that the flavor analyses of cooked potatoes were all based on GC-O-MS [21] or GC-MS [10,11,22,23] technologies. In combination with these two problems, an in-depth profile of volatile flavor compounds in steamed potatoes of six different varieties was sought by means of HS-GC-IMS in the present work. Through sensory evaluation, fingerprint establishment and principle component analysis (PCA), the differences in chemical substances in steamed potatoes from different varieties could be revealed. This study might provide an advisable method for potato flavor identification, and is expected to be of great significance in the development of breeding by impairing the off-flavor or improving the pleasant flavor.

## 2. Materials and Methods

### 2.1. Materials

In this study, the potato genotypes ‘Atlantic’ (S1), ‘Russet Burbank’ (S2), ‘Longshu No. 23’ (S3), ‘Longshu No. 7’ (S4), ‘Longshu No. 14’ (S5) and ‘Longshu No. 16’ (S6) that are prominently cultivated in northwest China were selected for experiments. All of these cultivarscultivated under the same environments and agricultural managements were harvested in October 2021 and provided by Gansu Academy of Agricultural Sciences. Prior to cooking, they were all stored at 4 °C until use. The average weight of each tuber from six varieties ranged from 180 to 350 g. The contents of dry matter, total starch and crude protein were approximately 24–27%, 16–21% and 2.1–3.7%, respectively (Table S1).

### 2.2. Potato Steaming Process

Similar sized and shaped potatoes from six varieties without damages were selected, washed with the running water and dried on the table. Then, the un-peeled potato tubers were cut in half and steamed in a pan with a food steamer at 100 °C for 40 min. After steaming, the potatoes samples of different varieties used for flavor analysis were peeled, drained and cooled at room temperature, and mashed to obtain a randomized sample. Eighteen tubers of each variety were prepared, nine of which were used for flavor detecting, and the others were used for sensory evaluating.

### 2.3. Sensory Evaluation

The sensory evaluation team was composed of 15 selected members (ages between 18 and 28), all of whom had been trained on a series of important aroma and taste compounds and texture traits. They were all familiar with the general descriptive analysis, and had sensory analysis experience of food products, including potato-based products. Evaluations took place in individual compartments, and the temperature was kept at 25 ± 1 °C. Three basic traits were selected, flavor, taste and texture, among which the flavor was the main focus before taste. The classification, definition and evaluative marks of magnitude 1 to 5 (1: weak, 3: medium, 5: strong) are listed in Table S2. The panelists need to wash their mouths before the next sample. The order of tasting samples was random and the obtained marks on this scale were measured and normalized to calculate the sensory profiles.

### 2.4. HS-GC-IMS Analysis

The volatile compounds of steamed potatoes from different varieties were separated and identified using a GC-IMS flavor analyzer (FlavourSpec^®^, Dortmund, Germany) according to the procedures reported by Yang et al. (2021) with some modification [24]. Briefly, 2 g samples of each variety were weighed and placed in 20 mL headspace glass vials, sealed with a matching lid and labeled. The samples were inserted into the sample tank at 60 °C for 15 min, and then an injection volume of 500 μL was injected by an injection needle at 85 °C for flavoring analysis.

#### 2.4.1. GC Parameters

A column (MXT-5, RESTEK, Bellefonte, PA, USA) (length of 15 m, inner diameter of 0.53 mm, membrane thickness of 1.0 μm) was used for separation by setting the chromatographic column temperature at 60 °C. A purity of N_2_ greater than 99.99% acted as the carrier gas, the flow rate of which was programmed as follows: 2 mL/min for 2 min in the beginning for separating difficult-to-separate substances at a lower rate, followed by linearly increasing to 100 mL/min over 2–20 min, so that the substances with different properties could be detected quickly via different flow rates. The whole running time was 20 min.

#### 2.4.2. IMS Parameters

The flow rate of drift gas was set as 150 mL/min, and the drift tube temperature was 45 °C. The tritium (^3^H) was the radioactive source of IMS for ionizing molecules, which led to a positive ionization mode. N-ketones C4–C9 were used to calculate the retention index (RI) of analytes with external references. Additionally, all the compounds were identified by comparing RI and the drift time that ions needed to reach the collector of standards in the NIST and IMS database.

### 2.5. Statistical Analysis

The volatile compounds in the steamed potato samples were analyzed using LAV (Laboratory Analytical Viewer)-Gallery Plot with three plug-ins (Reporter, Gallery plot and PCA) [25]. Each point in the analyzed spectra represented a kind of volatile chemical substance; the three-dimensional (3D) and two-dimensional (2D) spectra obtained by Reporter plug-in were used to intuitively compare the differences between six groups; the differences of a large number of compounds between different groups can be viewed by fingerprint obtained by Gallery plot plug-in; and the PCA plug-in was used for cluster analysis among various samples. Three replicates of steamed potatoes from each variety were conducted in the experiments and the data represent the mean ± SD; the significant difference was analyzed via Duncan’s test (*p* < 0.05). Origin 2021Pro and SPSS 26.0 software were used for chart making and statistical analyses.

## 3. Results and Discussion

### 3.1. Appearance Analysis and Sensory Evaluation of Steamed Potatoes from Six Varieties

**Figure 1:**
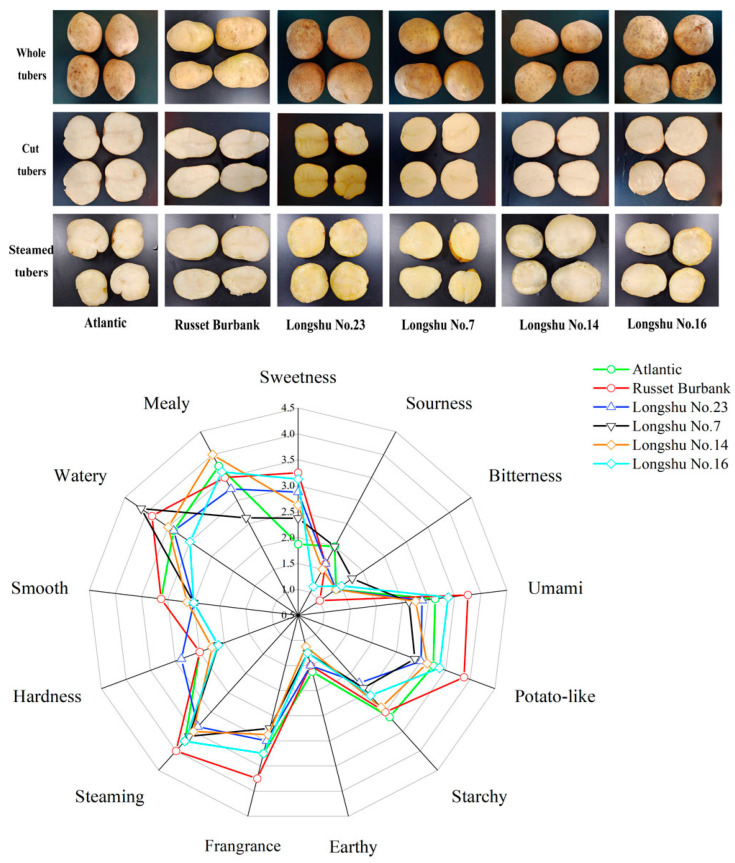
The appearance (whole, cut and steamed) and sensory evaluation of steamed potatoes from six varieties including Atlantic, Russet Burbank, Longshu No. 23, Longshu No. 7, Longshu No. 14 and Longshu No. 16.

Representative photographs of whole, cut and steamed potatoes from the different varieties are presented in Figure 1. The skins of all the varieties except for Russet Burbank were russeted and netted, and the flesh of Atlantic, Russet Burbank, Longshu No. 14 and Longshu No. 16 exhibited a white color, whereas another two varieties are yellowish. After the potatoes were steamed, they all maintained a good appearance without any breaking or pasting, which may be accounted for the lower solubilization of the serious nutrition and deconstruction of the cellular structure in the steamed potatoes in contrast with boiled potatoes [26].

The results of sensory evaluation showed that the variety Russet Burbank (S2) had the highest overall acceptability in taste, texture and flavor, followed by Longshu No. 16 (S6), but only in taste and flavor. However, the sourness and bitterness of six varieties all had lower scores, indicating that these varieties are suitable for steaming. The organic acids and glycoalkaloids in potatoes mainly contributed to sour and bitter tastes, respectively. After steaming or boiling, these substances would be further decreased, with higher edible quality [27]. Reducing sugars in the thermal reaction mainly resulted in a sweetness sensation; all the varieties had the similar scores in sweetness evaluation except for S1, with the lowest scores. The texture of potato is mainly controlled by dry matter, starch content and distribution, cell size and cell wall structure [13]. Results showed that the varieties S3, S2, S4 and S5 exhibited higher scores in hardness, smoothness, wateriness and mealiness, respectively. Flavor is closely related to the type and extent of volatile compounds released during cooking [28]. Our data showed that S2 and S6 obtained higher scores in flavor by evaluating them from the perspective of steaming, fragrance, earthy, starchiness and potato-like quality.

### 3.2. HS-GC-IMS Analysis

#### 3.2.1. HS-GC-IMS Topographic Plots of Steamed Potatoes from Different Varieties

**Figure 2:**
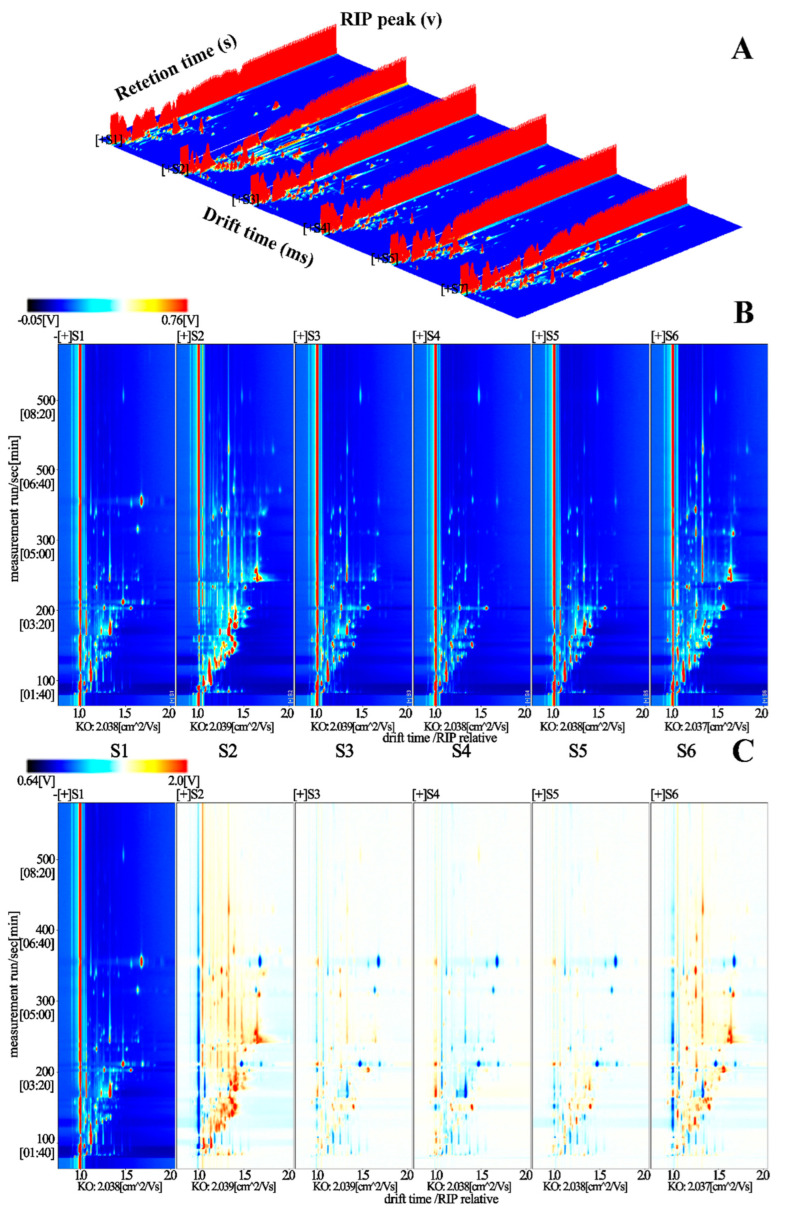
Volatile compounds in steamed potato samples of different varieties (from left to right: S1: Atlantic, S2: Burbank, S3: Longshu No.23, S4: Longshu No. 7, S5: Longshu No. 14, S6: Longshu No. 16) by HS-GC-IMS. (**A**) Three-dimensional topographic of volatile compounds of different varieties. (**B**) Two-dimensional spectrum of volatile components of different varieties (*x*-axis means ion migration time and y-axis means gas chromatography retention time). (**C**) Comparison of the difference spectrum of volatile components of different varieties.

It is well-known that there exist some volatiles in raw potatoes, but more typical volatiles would be produced when the potatoes are cooked [9]. HS-GC-IMS can be applied to find out about the global IMS profile from food samples, with the aim of identifying the flavoring chemical substances and the related mechanism in cooking [29]. In this study, the HS-GC-IMS was adopted to analyze the differences in volatile organic compounds of steamed potatoes of different varieties, and the final results can be displayed by the 3D topographic plot visualization in Figure 2A, where the transverse-, longitudinal- and vertical-axes refer to ion migration time, the retention time of gas chromatography and signal peak intensity, respectively. The 3D topographic map provides us with the original information of all the volatile compounds from steamed samples. By observing the 3D topographic plot, a similar visualization of steamed potatoes from different varieties was found, but the peak height between different groups showed slight differences.

In order to avoid the rough information of the 3D topographic plot and clearly distinguish each volatile compound detected in the steamed potato samples, the top view of the 2D topographic spectrum was further obtained by normalizing the ion migration time and reactive ion peak (RIP) position, which was shown in Figure 2B. The single point on the right side of RIP stands for volatile compounds extracted from the samples. Additionally, the colors displayed on the map refer to the signal peak strength of each substance; generally, red is a symbol of high concentration, whereas blue is the symbol of low concentration. It can be seen that most of the signal appeared at 100–350 s of retention time and 1–1.5 s of drift time. Through the top view of the 3D topographic spectrum, the compound contents of different species can be intuitively quantitated.

In the present work, the S1 (cv. Atlantic) was taken as a reference, so that the detailed compound information in other samples were deduced for effectively identifying the differences among six groups. After deduction, the background of two of the same volatile compounds is white, whereas the red points on the map represent a higher level than the reference and the blue ones represent a lower level. Figure 2C showed that the red plots in S2 were more than the reference, indicating that the volatile compound types were more abundant that S1. In addition, the concentration of S6 was also higher than the reference, which was followed by S2. Except for these two groups, the compounds from three other varieties showed similar and lower concentrations compared with S1. In general, the total concentration of flavor chemical substances in each potato sample differed significantly with the variety. In the S2 and S6 groups, the levels of most detected substances reached their highest after the potatoes were steamed. This may haveresulted from the maximum release of flavor substances in S2 and S6 after steaming.

#### 3.2.2. Qualitative Results of Volatile Compounds in Samples of Steamed Potatoes from Different Varieties

Although the topographic plots can provide some tendencies and information for detected substances, it is also difficult to accurately judge the volatile compounds on the map. Therefore, the fingerprint profiles of volatiles in steamed potatoes from different varieties were further generated by using LAV software. Each row and column of fingerprints represents the peak signal intensities of different volatiles from the same sample, and that of the same volatile compound from different varieties. A plot means a specific substance, where the darker the color is, the more abundant the substance is. As presented in Table 1, sixty-three types of volatile compounds, including twenty-seven aldehydes, fourteen alcohols, twelve ketones, four esters, two furans, one acid and others, were generated from six varieties. It was observed that there were some signals presenting as dimers or monomers due to a higher proton affinity of volatiles, or some adducts formation [30]. However, another fifteen substances were not identified by comparing with the IMS database.

The species and concentration of volatile compounds highly depend on the original nutrients in potatoes, including carbohydrates, free amino acids, fatty acids and a series of vitamins and minerals [9], which can be further transferred into multiple flavor compounds through thermal reaction, i.e., lipid oxidation, the Maillard reaction and Strecker degradation [10,11]. However, most of the aldehydes, ketones, alcohols, esters, furans and pyrazine were reported to be derived from the Maillard reaction or lipid degradation [12,19]. The total content of lipids, including phospholipids, glycolipids, galactolipids, sulpholipids and neutral lipids, occupied approximately 0.1–0.5% of the fresh weight of potato tubers [31,32]. On analyzing the results, the aldehydes mainly derived from lipid oxidation belonged to the key odor compounds in the steamed potatoes (Table 1), also suggesting that this type of compound had stronger volatility, higher levels and lower thresholds.

Alcohols are the main decomposition products of fat oxidation, which contributes significantly to the formation of the ideal flavor and aroma of potato [11]. The thermal oxidation brought about by cooking is the key reason for the generation of flavor. In this study, fourteen alcohols including 1-hexanol, 1-penten-3-ol, 2-methylbutanol, methionol, 1-octen-3-ol, 1-pentanol, 2-methylpropanol, ethanol and methanol were identified in steamed potatoes. The ketones are generally the principle oxidation products of unsaturated fatty acids, including linoleic and α-linolenic acid [2]. It was reported that the linoleic acid and linolenic acid contents in potato tubers were 52–60% and 13–24% of the total fatty acids, respectively [31]. In this study, a large proportion of ketones, such as 6-methyl-5-hepten-2-one, 2-heptanone, 3-hydroxy-2-butanone, 2-butanone, 3-octanone, 2-hexanone, 2-pentanone and acetone, were detected in six varieties. However, the oxidation of fatty acids is thought to be a major cause of an off flavor, and also to be related to the rancidity problem of foodstuffs.

Additionally, another class of detected volatiles are esters, which mainly arise from the esterification of alcohols and acids, generally endowing fruity flavors [33]. Our results showed that four esters, including ethyl acetate (D/M) and hexyl acetate (D/M), were detected in steamed potatoes. Furan is derived from the Maillard reaction, and produced in the process of heating; for example, 2-pentylfuran and 2-butylfuran It was reported that 2-pentylfuran is a non-carboxyl compound derived from linoleic acid with a lower threshold value [9]. Morris et al. (2010) demonstrated that these two furans were also detected in steamed potatoes of other cultivars [34]. Interestingly, the ortho-guaiacol was firstly identified in the steamed potatoes. It was reported that ortho-guaiacol might be closely related to the “spicy” and “smoky” descriptor in the Xinjiang dried figs [35]. In addition, the ortho-guaiacol was considered to be the main aromatic substance in steamed sorghum for brewing wine [36]. Therefore, this specific compound identified in this research may be another important contributor to the steamed potatoes. All of these volatiles were produced on the basis of a heating temperature over 100 °C. Therefore, most of the detected volatile compounds belonged to the common products of the thermal reaction during the steaming of potatoes.

#### 3.2.3. Fingerprint Analysis of Volatile Compounds in Steamed Potatoes of Different Varieties

**Figure 3:**
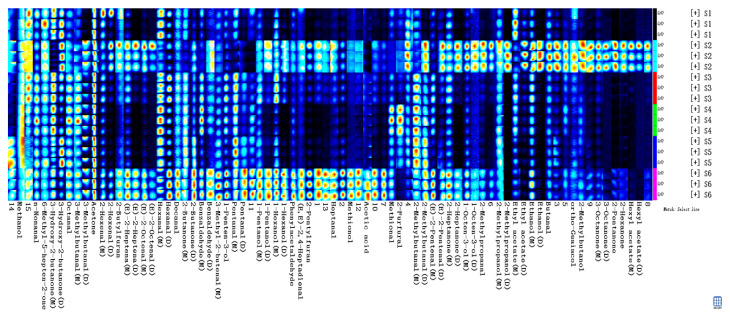
Gallery plot of the selected signal peak areas obtained with the steamed potato from different varieties (S1: Atlantic, S2: Russet Burbank, S3: Longshu No. 23, S4: Longshu No. 7, S5: Longshu No. 14, S6: Longshu No. 16), determined by HS-GC-IMS.

The information from the fingerprint showed that aldehydes, ketones, alcohols and esters make important contribution to the steamed potato flavor in the S2 group, of which the 2-methylbutanal, (E)-2-pentenal, 2-heptanone, 1-octen-3-ol, 2-methylpropanal, ethanol, butanal, ortho-guaiacol, 2-methyl butanal, 3-octanone, 2-pentanone, 2-hexanone, ethyl acetate and hexyl acetate in samples had the highest concentrations (Figure 3). Among these, 2-methylbutanal, 2-heptone, ethyl acetate and hexyl acetate had a fruity aroma and imparted a pleasant flavor [10,11,34]. As is consistent with sensory evaluation, S2 showed the highest scores in fragrance (Figure 1), indicating that these substances are the main contributors of fragrance. However, (E)-2-pentenal had a roasted, rubbery and unpleasant flavor [2], and 1-octen-3-ol imparts an earthy, mushroomy and rancid flavor [20,37]. In the S4 group, 2-furfural and methional showed the highest intensities compared with others. Methional is a kind of sulfur-containing compound, and is generally agreed to be a typical flavor in boiled potatoes, caused by the Maillard reaction [2,38].

A set of compounds such as acetic acid, methionol, heptanal, 2-pentylfuran, (E, E)-2, 4-heptadienal, phenylacetaldehyde, 1-hexanol, 1-pentanol, pentanal, 1-penten-3-ol, 3-methyl-2-butanenal, benzaldehyde, 2-butanone, decanal and hexanal showed brighter signals in the S6 group. The detected acetic acid made the steamed potato present a vinegary flavor [23]. It was reported that 2-pentylfuran showed unpleasant, green beans, cooked, rubber/cardboard-like off-odors and off-flavors [2,22,37], which were also detected in raw and boiled potatoes. The character-impact compounds presenting rancidness and off-flavor included (E, E)-2, 4-heptadienal, 1-pentanol and pentanal [37]. Concerning the phenylacetaldehyde, it imparted multiple flavor characteristics including green, flowery, earthy, honey, floral and sweet [23,39]. Interestingly, the phenylacetaldehyde was also reported to be the characteristic volatile compound in steamed sorghum [40]. Moreover, the decanal showed higher levels in S6 and imparted fruity, fatty, floral, burnt plastic and rancid flavors [10,11,37]. According to the sensory scores (Figure 1), S6 also had higher fragrance values, which may have resulted from these compounds. Gu et al. (2020) reported that decanal also has a roasted-note compound in the purple potatoes [41]. Additionally, hexanal represents green, grass, intense, savory, creamy and rancid flavors, and also had higher concentrations in contrast with other compounds in S6. It has been proved that high water temperatures of over 100 °C favor the production of hexanal, 2-heptenal, and lipid-derived compounds such as 2-methylfuran, 2-pentylfuran, 3-hexanone, and 1-octen-3-ol [30]. Meanwhile, we also found that volatile compounds including (E)-2-octenal, (E)-2-heptena, 2-butylfuran and 2-hexenal commonly had higher levels in S2 and S6 groups. (E)-2-octenal and (E)-2-heptena both had baked, boiled and fatty flavors [37,42], while 2-butylfuran could make potatoes possess sweetness [34]. All of these volatiles made S2 and S6 have a steamed characteristic (Figure 1). In addition, other volatile compounds in S2 and S6 group were also responsible for the pleasant odor or off-flavor, but these volatile metabolites could impair the sensory acceptability of steamed potatoes.

#### 3.2.4. Effect of Variety on the Volatile Compounds in Steamed Samples

**Figure 4:**
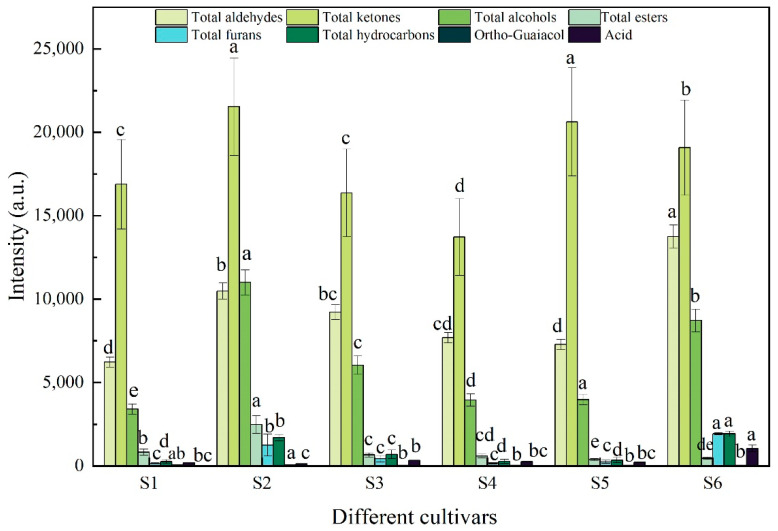
The total peak volume of the volatile flavor compounds in terms of aldehydes, ketones, alcohols, esters, furans acid and others detected in different varieties (S1: Atlantic, S2: Russet Burbank, S3: Longshu No. 23, S4: Longshu No. 7, S5: Longshu No. 14, S6: Longshu No. 16). All values were expressed as mean ± SD. Significant differences between different varieties via Duncan’s test were expressed by different small letters (*p* < 0.05).

The types and concentrations of potato flavor compounds were believed to be associated with the variety, cultivation conditions and processing methods, and show genotype specificity [36]. However, the variety may be the key factor that affects the flavor formatting and presentation. The fatty acids, sugars and amino acids vary greatly with-different varieties. Additionally, these substrates are the precursors of most compounds, including alcohols, aldehydes, ketones, esters and so on, which together contribute to flavor when the potato tubers are cooked [9]. The total peak intensities of aldehydes, ketones, alcohols, esters, furans, hydrocarbons and acid were calculated and are shown in Figure 4. The intensity of total ketones in different varieties showed maximum levels, followed by total aldehydes and total alcohols, suggesting that these three volatile compounds were the predominant components of the steamed potato flavor. This result is in accordance with the fingerprint analysis (Figure 3). Wang et al. (2019) also steamed the potatoes of six varieties and made them into mashed potatoes to evaluate the aroma components, and found that the proportion of alcohols, alkanes, aldehyde and furans were the greatest [43]. These similar results may indicate that the above typical compounds belong to the representative flavor substances in steamed potatoes. Beyond that, the intensities of other classes of compounds were lower. Moreover, statistical analysis showed that there was a distinct difference in volatile compounds within the six varieties. Large amounts of aldehydes, ketones and alcohols and trace amounts and numbers of others formed during the thermal reactions together had a greater effect on the flavor of steamed potatoes, which led to different tastes and flavors in different varieties after steaming.

### 3.3. Cluster Analysis (PCA) and Fingerprint Similarity Analysis of Flavor Compounds Based on Principal Component Analysis

**Figure 5:**
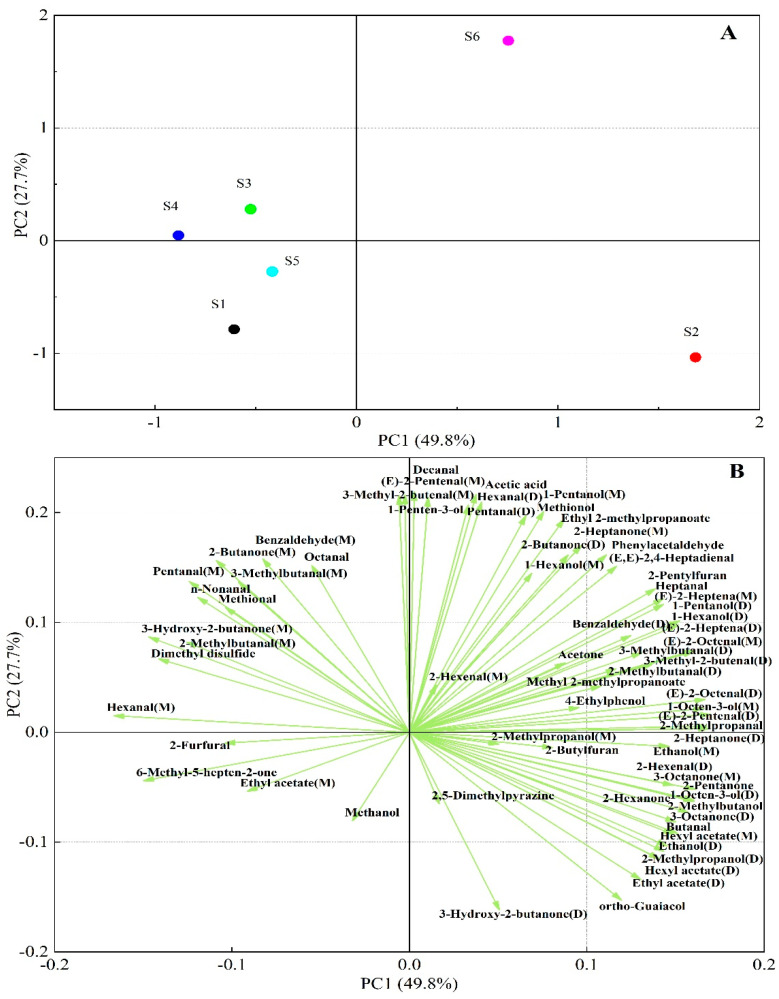
Principal component analysis in samples from steamed potatoes of six varieties (S1: Atlantic, S2: Russet Burbank, S3: Longshu No. 23, S4: Longshu No. 7, S5: Longshu No. 14, S6: Longshu No. 16). (**A**) Score plot of principal components; (**B**) loading plot of different variances.

Principal component analysis (PCA) is a multivariate statistical method used to determine the correlation between multiple variables, and is mainly measured by the signal strength of volatile substances so that the differences between samples can be highlighted [44]. PCA results, including the score plot and the loading plot of the volatile compounds in the steamed samples from different varieties, are shown in Figure 5. The first two principal components in the Figure were observed to make up 49.8% and 27.7% of the total variance contribution ration. The PCA model can be a preferred separation model if the cumulative contribution rate of PC1 and PC2 is more than 60% [45]. Therefore, our result of a total cumulative contribution of 77.5% indicated that it was sufficient enough to explain the similar characteristics (Figure 5A). The PCA score plot showed that the steamed potato samples of different varieties were distributed in relatively independent spaces, suggesting that the flavors were dramatically different among the six varieties. However, the samples of S1, S3, S4 and S5 are close on the map; they have similar flavor characteristics. Conversely, the distance between samples S2 and S6 was larger, indicating that the differences between them were more evident.

In order to better display the correlation between volatiles and samples, a loading plot was further analyzed, as described in Figure 5B. The sample S6 was mainly scattered in Ⅰ quadrant, which seemed to be positively corrected with a large number of aldehydes, alcohols and esters, such as 2-pentylfuran, 1-hexanol, 1-pentanol, pentanal, 1-penten-3-ol, 3-methyl-2-butenal, benzaldehyde (D), 2-butanone, (E)-2-pentenal and (E)-2-octenal, and so on. The samples of S3 and S4 (Ⅱ quadrant) were more characterized by 2-butanone, benzaldehyde (M), octenal, 3-mehtylbutanal (M), n-Nonanal, methional, 3-hydroxy-2-butanone (M) and 2-methylbutanal (M). Additionally, S1 and S5 samples that were correlated with 2-fufural, 6-methyl-5-hepten-2-one and ethyl acetate fell in the Ⅲ quadrant, whereas the notes of the S2 sample including 2-methylbutanal, 2-methyl propanal, ethyl acetate, ethanol, butanal, ortho-guaiacol, 2-methyl butanol, 3-octanone and hexyl acetate and so on, emerged in the Ⅳ quadrant. This result is greatly consistent with the fingerprint result. Hence, these data proved that the characterized flavor fingerprints of the steamed potato sample from different varieties were successfully established via HS-GC-IMS, which has given insight into the detailed information on flavor differences among different varieties and revealed the mechanisms of flavor substance formation in the potato steaming process.

## 4. Conclusions

This research focused on the determination of volatile compounds of steamed potatoes from six varieties. The sensory evaluation results showed that S2 and S6 samples had a rich flavor and led to a higher acceptability. Subsequently, a large number of flavor compounds were successfully identified after steaming. A total of 63 volatile chemical substances were identified across the steamed potatoes of six varieties, including aldehydes, alcohols, ketones, esters, furans, acid and others. The aldehydes, alcohols, ketones and esters were found to be the most abundant in S2 and S6, which may cause a more obvious flavor, including steaming, fragrance, potato-like and umami characteristics, than any other varieties. On the other hand, the intensity of the total ketones, aldehydes and alcohols in contrast with other compounds from all of the samples was the highest, suggesting that these compounds made an essential contribution to the flavor of steamed potatoes. The PCA uncovered that the steamed samples could be distinguished well in different varieties, especially S2 and S6. Hence, HS-GC-IMS is a promising method for understanding the effects of varieties on the volatile compound formation of cooked potatoes, and can also be used to improve or standardize the quality of the final steamed potato products.

## Figures and Tables

**Table 1:** GC-IMS global area set integration parameters obtained from the steamed potato samples.

Count	Compound	CAS#	Formula	MW ^a^	RI ^b^	Rt ^c^	Dt ^d^
Ketones							
1	6-Methyl-5-hepten-2-one	C110930	C_8_H_14_O	126.2	989.9	340.709	1.17549
2	2-Heptanone(M)	C110430	C_7_H_14_O	114.2	886.3	255.621	1.26628
3	2-Heptanone(D)	C110430	C_7_H_14_O	114.2	888	256.563	1.63635
4	3-Hydroxy-2-butanone(M)	C513860	C_4_H_8_O_2_	88.1	711.8	172.292	1.06848
5	3-Hydroxy-2-butanone(D)	C513860	C_4_H_8_O_2_	88.1	702.8	168.67	1.33287
6	2-Butanone(M)	C78933	C_4_H_8_O	72.1	582.1	131.616	1.06168
7	2-Butanone(D)	C78933	C_4_H_8_O	72.1	581.6	131.475	1.24727
8	3-Octanone(M)	C106683	C_8_H_16_O	128.2	989.3	340.099	1.3099
9	3-Octanone(D)	C106683	C_8_H_16_O	128.2	988.9	339.771	1.72054
10	2-Hexanone	C591786	C_6_H_12_O	100.2	771.7	198.396	1.4981
11	2-Pentanone	C107879	C_5_H_10_O	86.1	672.8	158.044	1.37331
12	Acetone	C67641	C_3_H_6_O	58.1	472.2	105.465	1.12323
Alcohols							
13	1-Hexanol(M)	C111273	C_6_H_14_O	102.2	865.1	243.988	1.32861
14	1-Hexanol(D)	C111273	C_6_H_14_O	102.2	866.4	244.701	1.64434
15	Ethanol(M)	C64175	C_2_H_6_O	46.1	418	94.528	1.0485
16	Ethanol(D)	C64175	C_2_H_6_O	46.1	421.2	95.15	1.13587
17	1-Octen-3-ol(M)	C3391864	C_8_H_16_O	128.2	982.1	333.275	1.15995
18	1-Octen-3-ol(D)	C3391864	C_8_H_16_O	128.2	983.4	334.565	1.60007
19	1-Pentanol(M)	C71410	C_5_H_12_O	88.1	756.6	191.436	1.25426
20	1-Pentanol(D)	C71410	C_5_H_12_O	88.1	754.3	190.412	1.51587
21	2-Methylpropanol(M)	C78831	C_4_H_10_O	74.1	621.7	142.563	1.17389
22	2-Methylpropanol(D)	C78831	C_4_H_10_O	74.1	623.9	143.203	1.37407
23	Methionol	C505102	C_4_H_10_OS	106.2	972.6	324.545	1.08599
24	Methanol	C67561	CH_4_O	32	397.6	90.724	0.98654
25	1-Penten-3-ol	C616251	C_5_H_1_0O	86.1	671	157.461	0.94444
26	2-Methylbutanol	C137326	C_5_H_12_O	88.1	723.7	177.201	1.48523
Aldehydes							
27	n-Nonanal	C124196	C_9_H_18_O	142.2	1104.9	503.956	1.47625
28	Phenylacetaldehyde	C122781	C_8_H_8_O	120.2	1043.3	407.761	1.25821
29	Methional	C3268493	C_4_H_8_OS	104.2	905.3	268.648	1.08682
30	2-Hexenal(M)	C505577	C_6_H_10_O	98.1	843.9	232.901	1.18075
31	2-Hexenal(D)	C505577	C_6_H_10_O	98.1	842.4	232.131	1.51787
32	Hexanal(M)	C66251	C_6_H_12_O	100.2	783	203.731	1.26665
33	Hexanal(D)	C66251	C_6_H_12_O	100.2	784.3	204.347	1.56356
34	(E)-2-Pentenal(M)	C1576870	C_5_H_8_O	84.1	741.4	184.712	1.10869
35	(E)-2-Pentenal(D)	C1576870	C_5_H_8_O	84.1	741.7	184.841	1.35597
36	(E)-2-Octenal(M)	C2548870	C_8_H_14_O	126.2	1058.1	429.033	1.33069
37	(E)-2-Octenal(D)	C2548870	C_8_H_14_O	126.2	1058.3	429.361	1.8206
38	2-Methylpropanal	C78842	C_4_H_8_O	72.1	555	124.625	1.28213
39	Octanal	C124130	C_8_H_16_O	128.2	1004.8	357.162	1.41166
40	Heptanal	C111717	C_7_H_14_O	114.2	898.4	263.467	1.68398
41	Benzaldehyde(M)	C100527	C_7_H_6_O	106.1	960.6	313.766	1.15051
42	Benzaldehyde(D)	C100527	C_7_H_6_O	106.1	959.7	312.96	1.46966
43	2-Methylbutanal(M)	C96173	C_5_H_10_O	86.1	655.7	152.67	1.16858
44	2-Methylbutanal(D)	C96173	C_5_H_10_O	86.1	660.7	154.206	1.40668
45	3-Methylbutanal(M)	C590863	C_5_H_10_O	86.1	642.2	148.576	1.18299
46	3-Methylbutanal(D)	C590863	C_5_H_10_O	86.1	639.2	147.681	1.40592
47	3-Methyl-2-butenal(M)	C107868	C_5_H_8_O	84.1	772.2	198.6	1.09275
48	(E,E)-2,4-Heptadienal	C4313035	C_7_H_10_O	110.2	1014.3	368.921	1.19433
49	2-Furfural	C98011	C_5_H_4_O_2_	96.1	822.2	222.074	1.08587
50	Butanal	C123728	C_4_H_8_O	72.1	596.8	135.571	1.29434
51	Pentanal(M)	C110623	C_5_H_10_O	86.1	685.1	162.01	1.19057
52	Pentanal(D)	C110623	C_5_H_10_O	86.1	689.3	163.417	1.42639
53	Decanal	C112312	C_10_H_20_O	156.3	1217.4	742.128	1.5427
Esters							
54	Ethyl acetate(M)	C141786	C_4_H_8_O_2_	88.1	604	137.549	1.09597
55	Ethyl acetate(D)	C141786	C_4_H_8_O_2_	88.1	602.4	137.125	1.33805
56	Hexyl acetate(M)	C142927	C_8_H_16_O_2_	144.2	1016.1	371.275	1.38917
57	Hexyl acetate(D)	C142927	C_8_H_16_O_2_	144.2	1016.9	372.26	1.90117
Acid							
58	Acetic acid	C64197	C_2_H_4_O_2_	60.1	612.8	140.008	1.05074
Furan							
59	2-Pentylfuran	C3777693	C_9_H_14_O	138.2	992.4	343.053	1.25272
60	2-Butylfuran	C4466244	C_8_H_12_O	124.2	889.9	257.673	1.17636
Others							
61	(E)-2-Heptena(M)	C18829555	C_7_H_12_O	112.2	955.1	309.008	1.25676
62	(E)-2-Heptena(D)	C18829555	C_7_H_12_O	112.2	956.2	309.936	1.67403
63	Ortho-Guaiacol	C90051	C_7_H_8_O_2_	124.1	1088	475.425	1.11647

^a^ represents molecular mass of compounds; ^b^ represents the retention index calculated using n-ketones C4–C9 as external references; ^c^ represents relative retention time in the capillary GC column; ^d^ represents the drift time in the drift tube. M and D mean monomer and dimer, respectively.

## Data Availability

Data is contained within the article or Supplementary Materials.

## Supplementary Materials

The following supporting information can be downloaded at: https://www.mdpi.com/article/10.3390/foods12112252/s1, Table S1: Nutrition components of fresh samples in different varieties (Atlantic, Russet Burbank, Longshu No. 7, Longshu No. 23, Longshu No. 14 and Longshu No. 16); Table S2: Scoring standards used for evaluating the samples of steamed potatoes from different varieties; The conditions of cultivation and fertilization of potatoes.
