# Supplementary material for: Development of a Flavor Fingerprint Using HS-GC-IMS for Volatile Compounds from Steamed Potatoes of Different Varieties
Source: Foods. 2023 Jun 2;12(11):2252. doi: 10.3390/foods12112252 (PMC10252913; doi:10.3390/foods12112252)
Supplement: Supplementary file 1 [file foods-12-02252-s001.zip › foods-2408620-supplementary.pdf]

Table. S1. Nutrition components including dry matter, starch, crude protein, vitamin C and reducing sugar of fresh samples in different varieties (Atlantic, Russet Burbank, Longshu No. 7, Longshu No. 23, Longshu No. 14 and Longshu No. 16)

Component / Variety	Dry matter (%)	Starch (%)	Crude protein (%)	Vitamin C (mg/100g)	Reducing sugar (%)
**Atlantic**	27.44	16.7	2.15	25.7	0.15
**Russet Burbank**	23.8	18.5	2.18	17.5	0.20
**Longshu No.7**	26	19.68	2.37	25.2	0.17
**Longshu No.23**	27	19.49	3.74	12.9	0.095
**Longshu No.14**	26.92%	20.24	2.63	16.23	0.19
**Longshu No.16**	26.9	21	2.68	9.8	0.23

Table. S2. Scoring standards used for evaluating the samples of steamed potatoes from different varieties.

Identified traits	Classification	Definition	Scores
**Taste**	Sweetness	A taste produced due to sugar contribution.	(1～5)
	Sourness	A taste produced by organic acids, such as chlorogenic, malic, and citric acids.	(1～5)
	Bitterness	A taste produced by some substances such as glycoside alkaloids, organic acids and total phenols.	(1～5)
	Umami	A taste produced by substances such as glutamate and aspartate.	(1～5)
**Flavor**	Potato-like	An aromatic that has raw vegetable-like character associated with peeled or uncooked potatoes.	(1～5)
	Starchy	The aromatics associated with starch and starch-based vegetables.	(1～5)
	Earthy	An aromatic that has an earthy character similar to fresh mushrooms.	(1～5)
	Fragrance	An aromatic that has fruity, flowery odor.	(1～5)
	Steamed	A moderately steamed impression that different from boiling.	(1～5)
**Texture**	Hard	Requiring a moderate amount of force to chew the sample.	(1～5)
	Smooth	Requiring a slight force when biting the sample.	(1～5)
	Watery	Perceiving amount of moisture in the sample when placed in the mouth.	(1～5)
	Mealy	Perceiving somewhat rounded smooth particles distributed within the sample.	(1～5)

The conditions of cultivation and fertilization:

The varieties used in present study were sowed in mid-April to mid-May and harvested in late September to late October. They are grown in the areas with rainfall of 400mm-600mm and cultivated by fertilizing 135kg pure nitrogen per hectare, 75 kg pure phosphorus per hectare, and 105 kg pure potassium per hectare.
